# Association of LEF1-AS1 with cardiovascular and neurological complications of COVID-19

**DOI:** 10.1016/j.jmccpl.2024.100280

**Published:** 2024-12-22

**Authors:** Mélanie Vausort, Andrew I. Lumley, Hassina Boubakeur, Lu Zhang, Feng Q. Hefeng, Markus Ollert, Paul Wilmes, Guy Fagherazzi, Yvan Devaux

**Affiliations:** a Cardiovascular Research Unit, Department of Precision Health, Luxembourg Institute of Health, Strassen, Luxembourg; b Bioinformatics and AI Unit, Department of Medical Informatics, Luxembourg Institute of Health, Strassen, Luxembourg; c Department of Infection and Immunity, Luxembourg Institute of Health, Esch-Sur-Alzette, Luxembourg; d Department of Dermatology and Allergy Center, Odense Research Center for Anaphylaxis (ORCA), University of Southern Denmark, Odense, Denmark; e Faculty of Science, Technology and Medicine, University of Luxembourg, Esch-sur-Alzette, Luxembourg; f Luxembourg Centre for Systems Biomedicine (LCSB), University of Luxembourg, Belvaux, Luxembourg; g Deep Digital Phenotyping Research Unit, Department of Precision Health, Luxembourg Institute of Health, Strassen, Luxembourg

**Keywords:** COVID-19, Biomarker, Complications, Cardiovascular, Neurological

## Abstract

A significant proportion of COVID-19 patients develop long-term complications, particularly cardiovascular and neurological issues. Even though risk factors for developing complications after COVID-19 have been identified, a biomarker to predict these complications could enable personalized healthcare and potentially reduce the disease burden. Easily measurable in the blood, the long noncoding RNA LEF1-AS1 has recently been associated with in-hospital mortality following SARS-CoV − 2 infection and holds potential as a biomarker for disease severity in COVID-19 patients. Consequently, we examined LEF1-AS1's ability to predict cardiovascular and neurological complications after COVID-19. LEF1-AS1 has been measured in the blood by quantitative PCR in 104 primo-infected participants from the Predi-COVID cohort within 3 days post clinical PCR-confirmed COVID-19 diagnosis. Among them, 35 participants (34 %) reported at least one persistent cardiovascular symptom and at least one persistent neurological or ocular symptom in a self-administered questionnaire 12 months after COVID-19 diagnosis. Blood levels of LEF1-AS1 at baseline in these patients were lower (*p* = 0.019) compared to those who did not report symptoms. Lower LEF1-AS1 levels were associated with symptoms with an odds ratio of 0.48 (95 % confidence interval 0.28–0.83) in a logistic regression model adjusted for age, sex, comorbidity, and moderate disease severity at baseline. LEF1-AS1 expression was positively correlated with the frequency of naïve T cells and negatively correlated with the frequency of effector memory T cells among total CD8+ T cells, revealing a potential association between LEF1-AS1 and CD8+ T-cell differentiation following SARS-CoV-2 infection. In conclusion, blood levels of LEF1-AS1 can potentially help in predicting 12-month cardiovascular and neurological complications in COVID-19 patients, though this finding requires validation in larger cohorts.

## Highlights

• Long term cardiovascular and neurological complications following SARS-CoV-2 infection are common.
• Long non-coding RNA LEF1-AS1 is reduced in the blood of COVID-19 patients with long-term complications.
• LEF1-AS1 expression is potentially associated with CD8+ T-cell differentiation following SARS-CoV-2 infection.
• Blood levels of LEF1-AS1 can potentially predict long term COVID-19 complications.

## Graphical abstract

**Figure:**
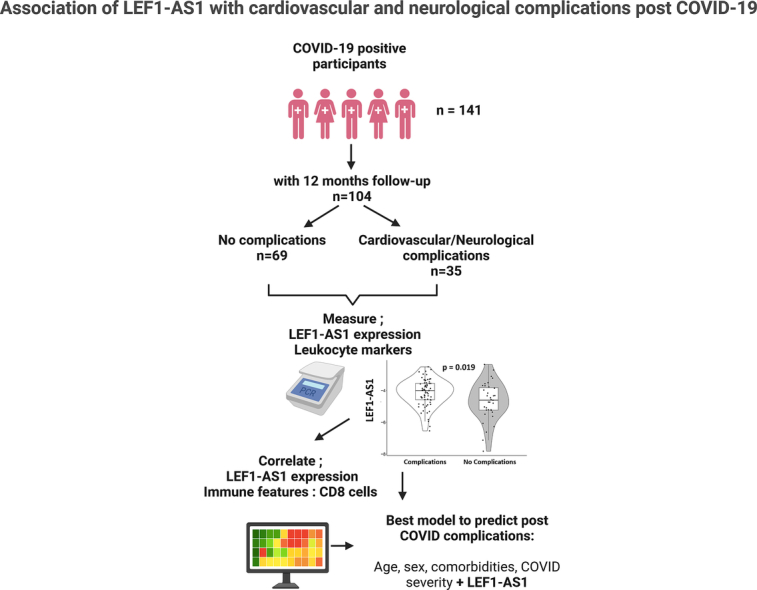

## 1 Introduction

In the wake of the COVID-19 pandemic, a critical concern has arisen regarding persistent health complications experienced by many individuals post-recovery. Beyond the acute phase of SARS-CoV-2 infection, a subset of patients present a broad range of symptoms including fatigue, respiratory issues, cognitive difficulties and/or cardiovascular complications, collectively referred to as post-acute COVID-19 syndrome or long COVID [1,2]. Indeed, significant proportions of patients report suffering from persistent, long-term cardiovascular and neurological issues after initial SARS-CoV-2 infection [3]. Estimating the number of people suffering from long-term (> 12 weeks) complications is challenging due to factors such as initial COVID-19 severity, age and sex. However, given the scale of the COVID-19 pandemic, even a low percentage of people suffering from long-term complications could represent millions of cases worldwide. As of 2024, therefore, COVID-19 and its knock-on effects continue to pose a significant economic burden and impact healthcare systems globally.

Recent studies have identified risk factors for long-term complications of COVID-19 which include, but are not limited to, demographic characteristics, age, female sex, comorbidities and the initial severity of the disease [[4], [5], [6], [7], [8]]. However, most of these findings predominantly rely upon self-reported data from questionnaires and surveys which, whilst lending insight, can often be biased. As such, easily detectable and measurable biomarkers for long-term complications following SARS-CoV-2 infection are greatly needed.

Not only serving as the basis of the SARS-CoV-2 vaccine, RNA molecules and their regulation have gained interest as clinical biomarkers over recent years for many diseases, including COVID-19 [[9], [10], [11], [12]]. The expression of Lymphoid Enhancer Binding Factor 1-antisense 1 (LEF1-AS1), for example, has recently been associated with in-hospital mortality following SARS-CoV-2 infection [13] and could be used as a biomarker of disease severity in COVID-19 patients [14].

Here, we investigated the ability of LEF1-AS1 to predict cardiovascular and neurological complications 12 months after COVID-19, and addressed associations with circulating immune cells.

## 2 Material and methods

### 2.1 Study population and end-point

The Predi-COVID study is a prospective cohort study conducted in Luxembourg, involving individuals aged 18 and above with a PCR-confirmed COVID-19 diagnosis [15]. The Predi-COVID study received approval from the National Research Ethics Committee of Luxembourg (Ethics Committee approvals 202,003/07 and 202,310/02-SU-202003/07) and was authorized by the Luxembourg Ministry of Health. Participants were recruited from April 2020 to December 2020 and provided blood samples at the latest 3 days post clinical PCR diagnosis. Blood samples were collected in the morning in PAXgene® RNA blood tubes (PreAnalytiX, Qiagen, Belgium) and were stored at −80 °c at the Integrated Biobank of Luxembourg. All participants reported experiencing a SARS-CoV-2 infection for the first time. Participants' demographic and clinical characteristics, including sex, age, BMI, and comorbidities, were collected at the time of inclusion. Any kind of pre-existing disease or condition was taken in consideration as comorbidity: hypertension, obesity, diabetes mellitus, chronic cardiac diseases, chronic pulmonary diseases (including asthma and chronic obstructive pulmonary disease), chronic kidney disease, liver disease, chronic neurological diseases (including dementia and multiple sclerosis), cancer, chronic blood diseases, HIV, rheumatic diseases and other disease conditions such as factor V Leiden, gastric reflux, Verneuil's disease, hyperthyroidism, burnout, hypercholesterolemia, hypotension, or vascular disease. The severity of acute COVID-19 disease was assessed according to NIH guidelines [16]. Participants were classified as asymptomatic, mild or moderate disease severity. Only 2 participants were hospitalized with a moderate disease severity at baseline. Among the 141 Predi-COVID participants used in [13], 104 have been enrolled in the present study since they were followed up after 12 months using a self-reported questionnaire to assess their general health status, persistent symptoms, and quality of life. The symptomatology at the 12-month mark has been categorized in previous studies in Predi-COVID [17,18]. The end-point of the present study was post-COVID-19 complications at 12 months, as indicated by the reporting by study participants of at least one persistent cardiovascular symptom (from the categories “Cardiorespiratory symptoms or diseases” or “Vascular/lymph node symptoms or diseases”) and at least one persistent neurological or ocular symptom after 12 months. The specific diseases included in each post-COVID-19 complication symptom have been outlined previously [18]. For Cardiorespiratory symptoms or diseases, these include shortness of breath, chest tightness, dry cough, fatty cough, tachycardia, arrhythmia, myocarditis, heart failure, burning chest, chest pain, wheezing, and coughing blood. For Vascular/lymph node symptoms or diseases, these include hypertension, hypotension, adenopathies, circulation disorders, and hematoma. For Neurological or ocular symptoms, these include tremors, headaches, migraines, mental confusion, malaise, convulsions, balance, memory, fatigue in eyes, hallucinations, sensitivity to light, and conjunctivitis.

### 2.2 RNA extraction and reverse transcription

PAXgene® RNA blood tubes were shipped to Firalis SA (Huningue, France) for RNA extraction in ISO 17025, ISO 9001, and CAP accredited facilities. Briefly, total RNA was extracted using the PAXgene® Blood miRNA kit (PreAnalytiX) according to the manufacturer's instructions. An on-column RNAse-free DNAse I step was performed. Total RNA concentration was assessed using the Qubit 3.0 fluorometer with the Qubit™ RNA High Sensitivity assay kit (Life Technologies, Belgium). Complementary DNA (cDNA) was synthesized from 400 ng of total RNA by using the High-Capacity RNA-to-cDNA™ Kit (Life Technologies) according to the manufacturers' instructions. Appropriate negative controls were performed to assess any genomic DNA contamination. cDNA samples were diluted 10 times in nuclease-free water prior to quantitative PCR.

### 2.3 Quantitative PCR

Diluted cDNA samples were used as template for quantitative PCR (qPCR). A 20 μl-reaction was performed in duplicate for each sample using the 2× IQ SYBR Green Supermix (Bio-Rad, Belgium). Specific primer pairs were designed with Beacon Designer software (Premier Biosoft, USA). Splicing Factor 3a Subunit 1 (SF3A1) was used as a housekeeping gene for normalization. Primer sequences, annealing temperature and qPCR efficiency are shown in Table S1. An inter-run calibrator was used to correct variations between different PCR plates. Expression levels were calculated by the relative quantification method (ΔΔCt) using the CFX Maestro 2.3 software (Bio-Rad).

### 2.4 Ex-vivo multicolour flow-cytometry-based deep immunophenotyping analysis

Blood samples collected in CPT tubes (Becton Dickinson, Belgium) at baseline were centrifuged for 20 min at 1800 x*g* at room temperature. The collected peripheral blood mononuclear cells (PBMCs) were washed twice in Ca^2+^-free PBS, counted using a Cellometer (Nexcelom, UK) and used for flow cytometry. 36 markers were used across 3 multicolor flow cytometry panels to analyze approximately 480 immune subsets/combinations in the PrediCOVID study, as reported in a previous study [19]. Deep immunophenotyping data were collected from 51 of the 104 PrediCOVID participants enrolled in the present study. This data was correlated with LEF1-AS1 expression measured in the same participants at baseline.

### 2.5 Statistical analysis

In R version 4.2.2, the packages lmtest, Hmisc, pROC, corrplot, and ggplot2 were used. Continuous variables were compared using two-sided unpaired Student's *t*-test or Wilcoxon test based on normality assessed by the Shapiro–Wilk test. Fisher's exact test or Chi-square test were used to compare proportions of categorical variables. Spearman's rank test was employed for correlation analysis. Univariate and multivariable logistic regression analyses assessed the association of variables with study end-point at 12 months. Each model's performance was evaluated using the AIC (Akaike information criterion) and the area under the receiver operating characteristic curve (AUC), along with the Wald test to determine the statistical significance. Odds ratios (OR) with 95 % confidence intervals (95 % CI) were calculated and visualized using a forest plot. Model selection criteria included a reduction in AIC and a significant Likelihood ratio test (Lr_*p*) for improved model fit. Integrated discrimination improvement (IDI) and net reclassification improvement (NRI), along with their corresponding *p*-values, were calculated to assess the improvement between 2 models. A *p*-value <0.05 was considered significant.

## 3 Results

### 3.1 Association between LEF1-AS1 and post-COVID-19 complications

The present study enrolled 104 patients of the Predi-COVID cohort who self-reported their persistent symptoms through a questionnaire after 12 months. Based on previous studies [17,18], we defined the end-point of the present study as post-COVID-19 complications, as indicated by participants reporting at least one persistent cardiovascular symptom (from the categories “Cardiorespiratory symptoms or diseases” or “Vascular/lymph node symptoms or diseases”) and at least one persistent neurological or ocular symptom, at 12 months after the initial COVID-19 infection. Out of the 104 participants, 34 % (35 participants) reported post-COVID-19 complications, while 66 % (69 participants) did not report any. The demographic and clinical characteristics of the 104 participants are presented in Table 1. The median age of the participants was 42.5 years, and 48.1 % were male. Participants who reported post-COVID-19 complications at 12 month follow-up had a significantly higher prevalence of at least 1 pre-existing comorbidity for any kind of disease (see list of diseases in the Methods section and in Table 1 footnote) compared to those who did not report complications (*p* < 0.001). Additionally, these participants experienced more severe forms of acute COVID-19 (*p* = 0.011).

**Table 1:** Demographic and clinical characteristics of study participants.

	All participants (*n* = 104)	No complication (*n* = 69)	CV/neuro complications (*n* = 35)	*p*-value (No vs CV/neuro complications)
Male, n (%)	50 (48.1)	37 (54)	13 (37)	0.112
Age, median (range)	42.5 (19–75)	41 (19–74)	46 (23–75)	0.180
BMI, median (range)	25 (19.0–39.9)	24.8 (19–37.6)	26.9 (19.1–34)	0.314
Comorbidity, n (%)	42 (40.4)	18 (26.0)	24 (69.0)	<0.001
Current smoker, n (%)	19 (18.3)	13 (19.0)	6 (17.0)	0.832
Former smoker, n (%)	22 (21.2)	14 (20.0)	8 (23.0)	0.762

Acute COVID-19 severity				
Asymptomatic, n (%)	16 (15.4)	13 (19.0)	3 (9.0)	0.011
Mild, n (%)	64 (61.5)	46 (67.0)	18 (51.0)	
Moderate, n (%)	24 (23.1)	10 (14.0)	14 (40.0)	

All variables were recorded during the acute phase of COVID-19, except for post-COVID-19 complications assessment, which was conducted after 12 months. Comorbidity can arise from any of the following diseases: hypertension, obesity, diabetes mellitus, chronic cardiac diseases, chronic pulmonary diseases (including asthma and chronic obstructive pulmonary disease), chronic kidney disease, liver disease, chronic neurological diseases (including dementia and multiple sclerosis), cancer, chronic blood diseases, HIV, rheumatic diseases and other disease conditions such as factor V Leiden, gastric reflux, Verneuil's disease, hyperthyroidism, burnout, hypercholesterolemia, hypotension, or vascular disease. The Wilcoxon or *t*-test was used to compare continuous variables between patients who declared at least one cardiovascular and one neurological complication as compared to patients who did not (No vs CV/neuro complications). Chi-square test was used to compare proportions. A *p*-value of <0.05 was considered significant and is highlighted in bold.

**Fig. 1:**
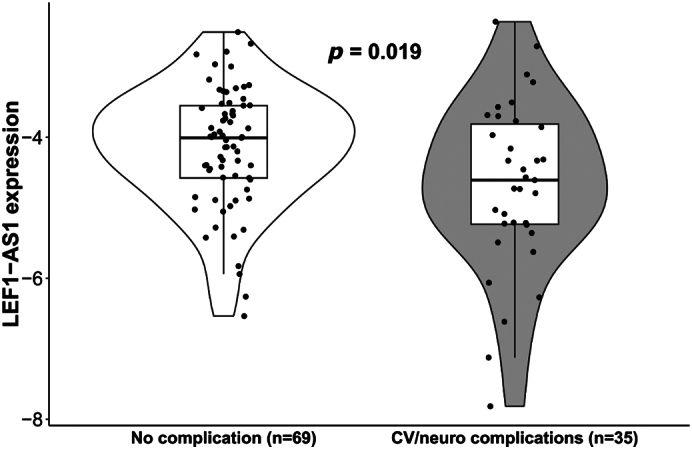
Expression of LEF1-AS1 according to post-COVID-19 complications. The expression level of LEF1-AS1 was measured by qPCR using total RNA extracted from PAXgene RNA blood tubes collected at baseline. LEF1-AS1 expression was Log2 transformed and normalized using SF3A1. The 104 participants were divided into two groups: 35 participants with persistent cardiovascular and neurological complications 12 months after COVID-19 and 69 participants without complication. The Wilcoxon test was used to compare the two groups. A *p*-value of <0.05 was considered significant and is highlighted in bold.

**Fig. 2:**
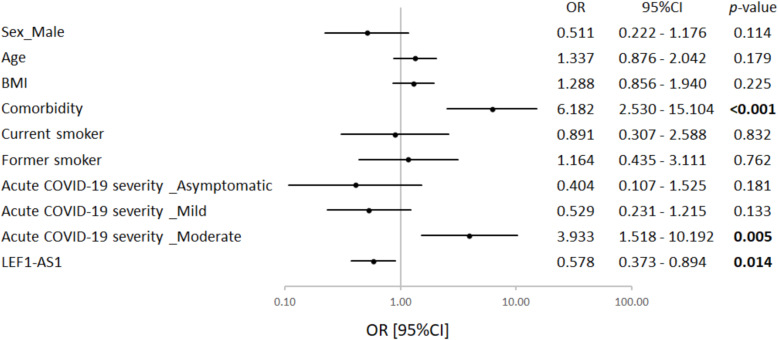
Univariate logistic regression analysis to predict post-COVID-19 complications. The forest plot displays the odds ratio (OR) with a ± 95 % confidence interval (95 % CI) and the corresponding *p*-values for each variable. A p-value <0.05 is considered significant and is highlighted in bold.

The expression of LEF1-AS1 was measured in the blood by qPCR during the acute phase of COVID-19 (baseline). LEF1-AS1 expression showed no association with the time or day of blood collection (Fig. S1A–B). Participants reporting post-COVID-19 complications exhibited lower LEF1-AS1 expression compared to those who did not (Fig. 1; *p* = 0.019). Using univariate logistic regression, having at least 1 pre-existing comorbidity and having experienced a moderate acute COVID-19 were associated with a 6-fold and 4-fold increased risk of having cardiovascular or neurological symptoms at 12 months, respectively (Fig. 2). Interestingly, LEF1-AS1 expression was associated with a reduced risk of post-COVID-19 complications (OR [95 % CI] 0.578 [0.373–0.894]).

**Fig. 3:**
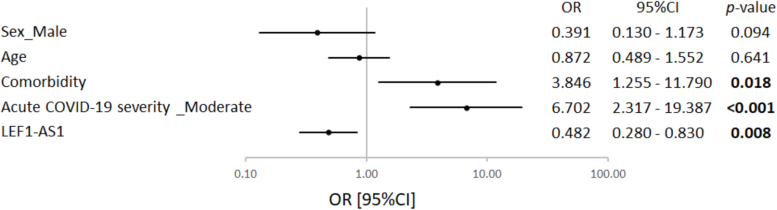
LEF1-AS1 as an independent predictor of post-COVID-19 complications. Multivariable logistic regression assessed associations between sex, age, comorbidity, acute severity and LEF1-AS1 expression with post-COVID-19 complications. All variables were recorded during acute COVID-19, except for post-COVID-19 complications assessment. The forest plot displays the odds ratio (OR) with a ± 95 % confidence interval (95 % CI) and the corresponding *p*-values for each variable. A *p*-value <0.05 is considered significant and is highlighted in bold.

In a multivariable model (Table 2), adding LEF1-AS1 along with pre-existing comorbidities and moderate acute COVID-19 significantly enhanced its association with the risk of cardiovascular and neurological complications (Akaike Information Criterion (AIC) of 110.428 for the model with LEF1-AS1 vs 114.354 without LEF1-AS1, *p* = 0.015). The full model with LEF1-AS1 had a predictive capacity with an AUC of 0.827 (Table 2). Furthermore, the inclusion of LEF1-AS1 to the model led to a significant reclassification of participants who were misclassified by the model without LEF1-AS1, as attested by a net reclassification improvement of 0.590 (*p* = 0.003) and an integrated discrimination improvement of 0.053 (*p* = 0.031). This model, which includes comorbidity, moderate acute COVID-19, and LEF1-AS1, demonstrated similar performance even after adding sex and age (AIC = 110.901, *p* = 0.171). In the sex and aged-adjusted model, comorbidity, moderate acute COVID-19, and LEF1-AS1 conserved their significant association with post-COVID-19 complications (Fig. 3). This supports an independent predictive value of LEF1-AS1 and suggests that participants with lower baseline levels of LEF1-AS1 in their blood at the time of infection were more likely to experience post-COVID-19 cardiovascular and neurological complications (OR [95 % CI] 0.482 [0.280–0.830]). The sex and age-adjusted model with comorbidity, moderate acute COVID-19 and LEF1-AS1 was selected as the reference model for this study.

**Table 2:** Multivariable logistic regression analysis to predict post-COVID-19 complications.

	AIC	AUC	Wald_p	Lr_p	NRI	NRI_p	IDI	IDI_p
Comorbidity + moderate acute COVID-19 severity	114.354	0.760	1.58E-04	–	–	–	–	–
+ LEF1-AS1	110.428	0.800	1.67E-04	0.015	0.590	0.003	0.053	0.031
+ LEF1-AS1 + sex (male) + age	110.901	0.827	0.001	0.171*	0.359*	0.077*	0.023*	0.178*

AIC: Akaike Information Criterion, AUC: area under the curve, Wald_p: Wald *p*-test, Lr_p: likelihood ratio test *p*-value, NRI: net reclassification improvement, NRI_p: net reclassification improvement *p*-value, IDI: integrated discrimination improvement, IDI_p: integrated discrimination improvement *p*-value. A *p*-value of <0.05 was considered significant and is highlighted in bold. *Comparison with the model composed by comorbidity, moderate acute COVID-19 severity and LEF1-AS1.

### 3.2 Association between leucocyte markers and post-COVID-19 complications

**Fig. 4:**
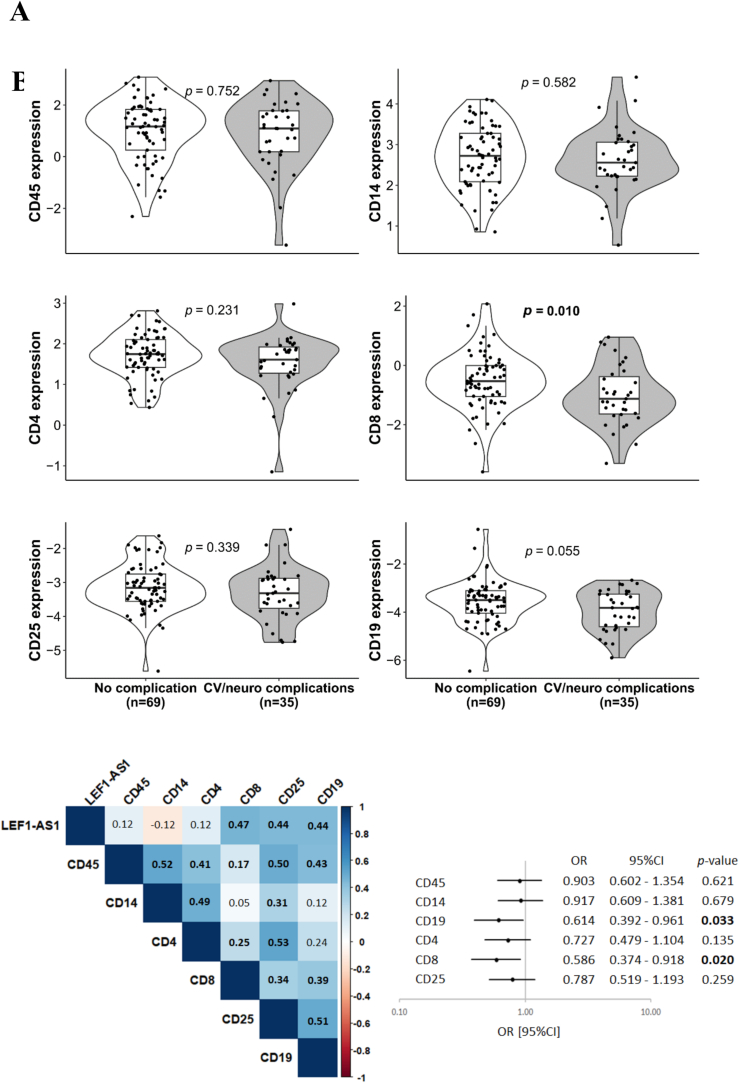
Expression and predictive value of leukocyte markers in relation to post-COVID-19 complications. The expression level of leukocyte markers (CDx) and LEF1-AS1 were measured by qPCR using total RNA extracted from PAXgene RNA blood tubes collected at baseline. Their expression was normalized using SF3A1 and log2-transformed. (A) The Wilcoxon or *t*-test was used to compare continuous variables between the two groups. (B) Spearman's correlation coefficients (R) are shown between LEF1-AS1 and leukocyte markers. (C) Univariate logistic regression to predict post-COVID-19 complications. The forest plot displays the odds ratio (OR) with a ± 95 % confidence interval (95 % CI) and the corresponding *p*-values for each leukocyte marker. A *p*-value <0.05 is considered significant and is highlighted in bold.

As previously mentioned, LEF1-AS1 expression was measured in blood samples from Predi-COVID participants. LEF1-AS1 is known to regulate the LEF1 gene and is involved in the development and function of immune cells, particularly lymphocytes [20]. Therefore, we investigated the association between different leukocyte subtypes and post-COVID-19 complications. Using qPCR, we measured the expression of six leukocyte markers in the same 104 blood samples used to measure LEF1-AS1: CD45 for all leukocytes, CD14 for monocytes, CD19 for B cells, CD4 for helper T cells, CD8 for cytotoxic T cells, and CD25 for activated T cells. The expression levels of these markers are presented according to post-COVID-19 complications at 12 months (Fig. 4A). The expression levels of CD45, CD14, CD4, and CD25 were similar in participants with or without complications. However, CD8 expression was significantly lower in participants who declared post-COVID-19 complications (*p* = 0.010).

Additionally, correlation analyses between LEF1-AS1 and these markers (Fig. 4B) showed moderate significant correlations with CD8, CD19, and CD25 (*R* = 0.47, 0.44, and 0.44, respectively), suggesting that LEF1-AS1 may be up-regulated in these leukocyte subtypes in participants with complications after COVID-19. However, only CD8 and CD19 were significantly associated with complications, with odds ratios of 0.586 [0.374–0.918] and 0.614 [0.392–0.961], respectively, in univariate logistic regression (Fig. 4C).

In multivariable logistic regression (Table 3), we assessed the incremental predictive value of each leukocyte marker to the reference model (including comorbidity, moderate acute COVID-19 severity, LEF1-AS1, sex and age) for predicting post-COVID-19 complications. The addition of any leukocyte marker significantly enhanced the predictive value of the reference model, as supported by the absence of a decrease in AIC and a significant likelihood ratio test. These results suggest that LEF1-AS1 retained its independent predictive value for identifying post-COVID-19 complications in the reference model, even after adjustment with leukocyte markers.

**Table 3:** Association between leukocyte marker expressions and post-COVID-19 complications (multivariable logistic regression).

	AIC	AUC	Wald_p	Lr_p*	NRI*	NRI_p*	IDI*	IDI_p*
Comorbidity + moderate acute COVID-19 severity + LEF1-AS1 + sex (male) + age	110.901	0.827	0.001	–	–	–	–	–
+ CD45	112.889	0.825	0.002	0.912	0.128	0.533	-2.65E-06	0.998
+ CD14	111.900	0.829	0.001	0.317	0.301	0.140	0.006	0.506
+ CD19	109.108	0.845	0.001	0.051	0.448	0.027	0.026	0.176
+ CD4	111.658	0.831	0.001	0.265	0.133	0.515	0.009	0.456
+ CD8	112.298	0.827	0.001	0.437	0.014	0.946	0.004	0.609
+ CD25	112.894	0.825	0.002	0.931	−0.012	0.952	2.69E-04	0.754

AIC: Akaike Information Criterion, AUC: area under the curve, Wald_p: Wald *p*-test, Lr_p: likelihood ratio test *p*-value, NRI: net reclassification improvement, NRI_p: net reclassification improvement *p*-value, IDI: integrated discrimination improvement, IDI_p: integrated discrimination improvement *p*-value. A *p*-value of <0.05 was considered significant and is highlighted in bold. *Comparison with the model composed by comorbidity, moderate acute COVID-19 severity, LEF1-AS1, sex (male) and age.

### 3.3 Association between LEF1-AS1 and immune cells by deep immunophenotyping

**Fig. 5:**
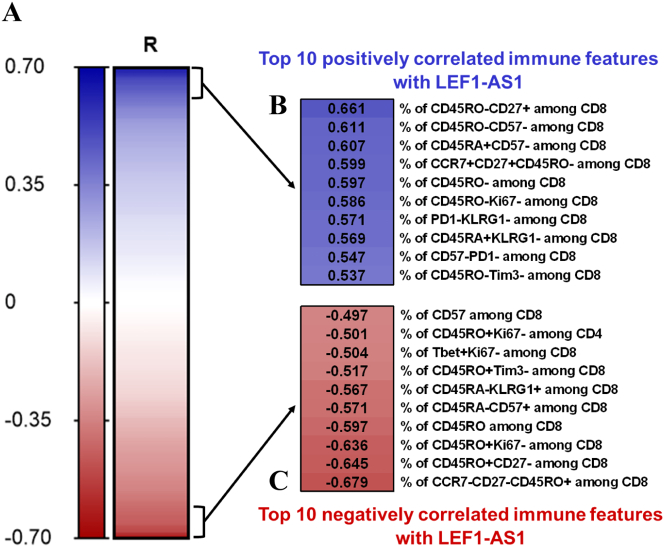
Correlation between LEF1-AS1 and immune features. 37 study participants were included in these analyses. All variables were recorded during the acute phase of COVID-19. The expression level of LEF1-AS1 was measured by qPCR using total RNA extracted from PAXgene RNA blood tubes collected at baseline and normalized using SF3A1. Flow cytometry was performed on fresh PBMCs to investigate 484 cellular immune subsets or combinations of various lineage and functional markers, as described previously [19]. (A–C) Spearman's correlation was performed between LEF1-AS1 and the 484 immune features. Heat map shows correlation coefficients, as signified by the ‘R’, for 484 immune features (A). Panels (B) and (C) display a zoom from (A) on the 10 immune features that have the strongest positive and negative correlations with LEF1-AS1, respectively, all with *p*-values <0.001.

To further explore the relationship between LEF1-AS1 and immune cell subtypes, correlation analyses were conducted between the expression levels of LEF1-AS1 and 484 immune features obtained by staining fresh PBMCs collected at baseline using 36 markers across 3 multicolor flow cytometry panels [19] (Fig. 5A). LEF1-AS1, deep immunophenotyping data and 12-month follow-up data were available for 37 study participants. Among the 484 immune features, 109 showed weak to moderate correlations with LEF1-AS1 (*p* < 0.05): 47 were positively correlated with correlation coefficients (R) ranging from 0.325 to 0.661, and 62 were negatively correlated with R values ranging from −0.327 to −0.679. The 10 most positively and negatively correlated immune features with LEF1-AS1 are presented in Fig. 5B and C, respectively. LEF1-AS1 showed predominant association with subpopulations of CD8+ cells in the baseline blood samples. The strongest positive correlation was observed between the percentage of CD45RO-CD27+ cells among CD8+ T cells, representing naïve T cells (*R* = 0.661, *p* < 0.001). Additionally, the percentage of CCR7 + CD27 + CD45RO- cells among CD8+ T cells, another marker for naïve T cells, also showed a positive correlation with LEF1-AS1 (*R* = 0.599, p < 0.001). Conversely, the strongest negative correlation was observed with the percentage of CCR7-CD27-CD45RO+ cells among CD8+ T cells, representing effector memory T cells (*R* = −0.679, p < 0.001). The distributions of the 10 most positively and negatively correlated immune features with LEF1-AS1, according to the outcomes after COVID-19, are plotted in Fig. S2A–B. None of the tested features were significantly deregulated between participants with or without post-COVID-19 complications.

## 4 Discussion

The study on complications following COVID-19 provides valuable insights into the disease's long-term impact. In addition to psychological impact and fatigue, a range of cardiovascular, respiratory and neurological complications have been identified, significantly affecting quality of life and overall health [21]. Understanding the mechanisms behind persistent symptoms post-COVID-19 is crucial, and developing biomarkers is essential for early detection and effective management of affected patients. Following our recent finding that blood levels of LEF1-AS1 predict COVID-19 disease severity and in-hospital mortality [13,14], we hereby investigated the ability of LEF1-AS1 to predict persistent symptoms after COVID-19.

Among the 104 participants of the longitudinal Predi-COVID cohort enrolled in this study, 34 % of individuals reported as experiencing at least one cardiovascular or respiratory symptom, and at least one neurological or ocular symptom at 12-month follow-up. The median age of the participants was 42.5 years, considered as middle-aged adults, and with an almost equal distribution of males and females. Interestingly, the median ages of participants with or without complications were similar (41 vs. 46, *p* = 0.180), indicating that age may not be a determinant of a poor outcome in this study population. Participants declaring symptoms at follow-up were more likely to have had pre-existing comorbidities and more severe acute COVID-19. This aligns with previous studies that also identified baseline comorbidities and disease severity as predictors of long-term complications [[4], [5], [6], [7], [8]].

In our study, LEF1-AS1 expression was found to be significantly lower in participants with a poor outcome compared to those with a good outcome. Using both univariate and multivariable logistic regression, we observed that having at least a pre-existing comorbidity, having experienced a moderate acute COVID-19 and having low levels of LEF1-AS1 at baseline were significantly associated with the risk of developing cardiovascular and neurological complications after COVID-19. Completed with calculations of reclassification indexes and the Akaike Information Criterion, these analyses revealed that LEF1-AS1 holds an independent predictive value. Even though sex and age were not significantly associated with the end-point in our study, and since other studies have highlighted that females are more susceptible to post COVID-19 syndrome and that age is a significant risk factor for developing the condition [8,22,23], we have chosen to keep sex and age in the reference model to enhance its applicability for future research.

Immune dysregulation is central to SARS-CoV-2 infection and results in a distinctive pattern of blood leukocytes. As the disease severity increases, there is a proportional reduction in lymphocytes, monocytes, basophils, and eosinophils [24,25]. Lymphopenia is one of the common features of COVID-19 and the degree of lymphopenia predicts disease severity [26,27]. Since LEF1-AS1 was measured in blood during the acute phase of COVID-19 in our study, we examined the association between leukocyte levels and LEF1-AS1. The analysis of leukocyte marker expression levels revealed that CD8, a marker for cytotoxic T cells, was significantly lower in participants with post-COVID-19 complications, similarly as observed for LEF1-AS1. The correlation analysis between LEF1-AS1 and these markers supports the hypothesis that LEF1-AS1 might be regulated in CD8+ and CD19+ cells. Deep immunophenotyping further elucidated the association between LEF1-AS1 and various immune features, predominantly CD8+ cell subpopulations during the acute phase of COVID-19. LEF1-AS1 was positively correlated with naïve T cells (% of CD45RO-CD27+ and CCR7 + CD27 + CD45RO- among CD8 cells) and negatively correlated with effector memory T cells (% of CCR7-CD27-CD45RO+ cells among CD8+ T cells). These observations suggest that LEF1-AS1 could play a role in the differentiation process in CD8+ cells after SARS-CoV-2 infection, consistent with the known role of LEF1-AS1 in the generation of memory precursors and functional memory CD8+ T cells [20]. Despite the correlations, none of these immune features showed a differential regulation between outcome groups at follow-up. This indicates that while LEF1-AS1 is associated with certain immune cell characteristics, its role in post-COVID-19 outcomes may not be solely dependent on cell counts but could involve other functional mechanisms within CD8+ cells.

A limitation of this study is the small sample size. While the findings are promising, the low number of participants may limit the generalizability of the results. Moreover, the self-reported nature of symptom data introduces potential bias, as subjective reporting can vary greatly among individuals. Also, almost two-thirds of patients who reported cardiovascular or neurological complications at follow-up also declared having comorbidities at the time of COVID-19 diagnosis, limiting the strength of the association between LEF1-AS1 levels and the de novo development of complications post COVID-19. Following on from this, the potential influence of other variables such as age, pre-existing comorbidities, medication and disease severity on LEF1-AS1 expression and it's correlation with immune markers is lacking from this study. Further investigation of the potential of LEF1-AS1 to be used as a predictive marker for long-term complications post COVID-19 should take these variables into account to strengthen the robustness of our findings. Moreover, the expression of LEF1-AS1 should be further examined in non-disease states (healthy individuals) to gain more knowledge on basal expression levels. This work is vital to establish a scale of expression before being able to use LEF1-AS1 in a clinical setting as a predictive biomarker. Despite these limitations, LEF1-AS1 remained a significant predictor even after adjustment with baseline comorbidities. To validate these findings, it is essential to conduct similar studies in larger cohorts.

In conclusion, the study identifies LEF1-AS1 as a potential biomarker for predicting long-term cardiovascular and neurological complications post-COVID-19. This biomarker could help guiding the development of targeted interventions for patients at risk of persistent symptoms post-COVID-19.

## Ethics declarations

Ethics approvals: The Predi-COVID and CoVaLux studies were approved by the National Research Ethics Committee of Luxembourg (Ethics Committee approvals 202,003/07 and 202,310/02-SU-202003/07) and were authorized by the Luxembourg Ministry of Health.

## CRediT authorship contribution statement

**Mélanie Vausort:** Writing – original draft, Formal analysis. **Andrew I. Lumley:** Writing – original draft. **Hassina Boubakeur:** Formal analysis. **Lu Zhang:** Formal analysis. **Feng Q. Hefeng:** Resources. **Markus Ollert:** Resources. **Paul Wilmes:** Resources. **Guy Fagherazzi:** Resources. **Yvan Devaux:** Writing – review & editing, Supervision, Project administration, Conceptualization.

## Funding

This work was supported by the Luxembourg Government through the CoVaLux programme (16954531).

Y.D. is funded by the EU Horizon 2020 project COVIRNA (grant agreement # 101016072), the 10.13039/501100001866National Research Fund (grant # COVID-19/2020-1/14719577/miRCOVID), the 10.13039/501100004562Ministry of Higher Education and Research, and the Heart Foundation-Daniel Wagner of Luxembourg.

## Declaration of competing interest

Y.D. holds patents and licensing agreements related to the use of RNAs for diagnostic and therapeutic purposes, and is member of the Scientific Advisory Board of Firalis SA.

## Appendix A Supplementary data

Supplementary material Image 1
