# Supplementary material for: Association of LEF1-AS1 with cardiovascular and neurological complications of COVID-19
Source: J Mol Cell Cardiol Plus. 2024 Dec 22;11:100280. doi: 10.1016/j.jmccpl.2024.100280 (PMC11967013; doi:10.1016/j.jmccpl.2024.100280)
Supplement: Supplementary file 1 — Supplementary material [file mmc1.docx]

**Association of LEF1-AS1 with cardiovascular and neurological complications of COVID-19.**

By Vausort et al.

**SUPPLEMENTARY INFORMATION**

**Table S1. Primer pairs used in qPCR.**

| **Target** | **Accession number** | **Forward sequence**  **(5’-3’)** | **Reverse sequence**  **(5’-3’)** | **Annealing temperature (°C)** | **qPCR efficiency (%)** |
| --- | --- | --- | --- | --- | --- |
| **SF3A1** | NM_005877 | GATTGGCCCCAGCAAGCC | TGCGGAGACAACTGTAGTACG | 60 | 103.9 |
| **LEF1-AS1** | ENST00000506314.1 | GTCCATGCTATGACCATCTCCA | ACACGAGTTAAGGCACATTCAC | 60 | 102.6 |
| **CD45** | NM_080921 | GTGCTTTGTTAAATCTCT | TATCTTCTTGATGGTTGT | 58 | 111.3 |
| **CD14** | NM_000591 | TAAAGCACTTCCAGAG | AATCTTCATCGTCCAG | 56 | 95.0 |
| **CD19** | NM_001770 | CTCAAGACGCTGGAAAGTAT | ACAGGCAGAAGATCAGATAAG | 60 | 97.4 |
| **CD4** | NM_000616 | AGTATGCTGGCTCTGGAA | CTGAGTGGCTCTCATCAC | 58 | 94.8 |
| **CD8(B)** | NM_004931 | TTCATTCTCAATCTCACAAG | GAAATCAACCACACTCAG | 58 | 105.0 |
| **CD25** | NM_000417 | GCGGAGACAGAGGAAGAG | GACCATTTAGCACCTTTGATTTC | 60 | 96.6 |


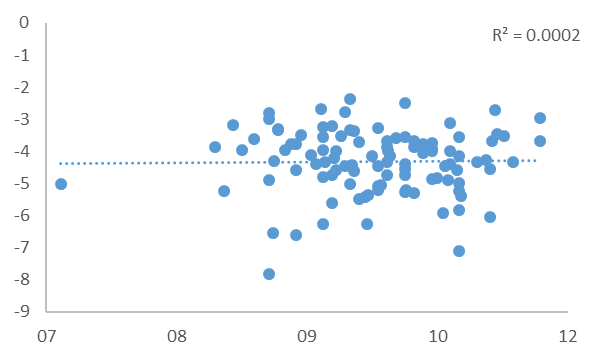


**LEF1-AS1 expression**

**Time of day (h)**

**A**

**B**


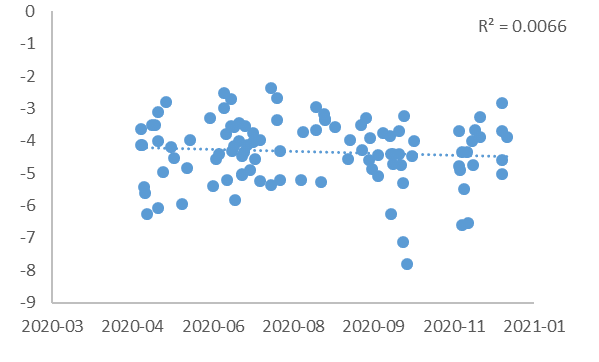


**LEF1-AS1 expression**

**Date of blood collection (day)**

**Figure S1. Assessment of the impact of blood collection time (A) and date (B) on LEF1-AS1 expression.** The expression level of LEF1-AS1 was measured by qPCR using total RNA extracted from PAXgene RNA blood tubes collected at baseline from 104 participants. LEF1-AS1 expression was normalized using SF3A1 and log-2 transformed. The normalized LEF1-AS1 expression levels were then plotted against the time of day (A) and the date of blood collection (B). The R² value was calculated to assess the strength of the correlation.


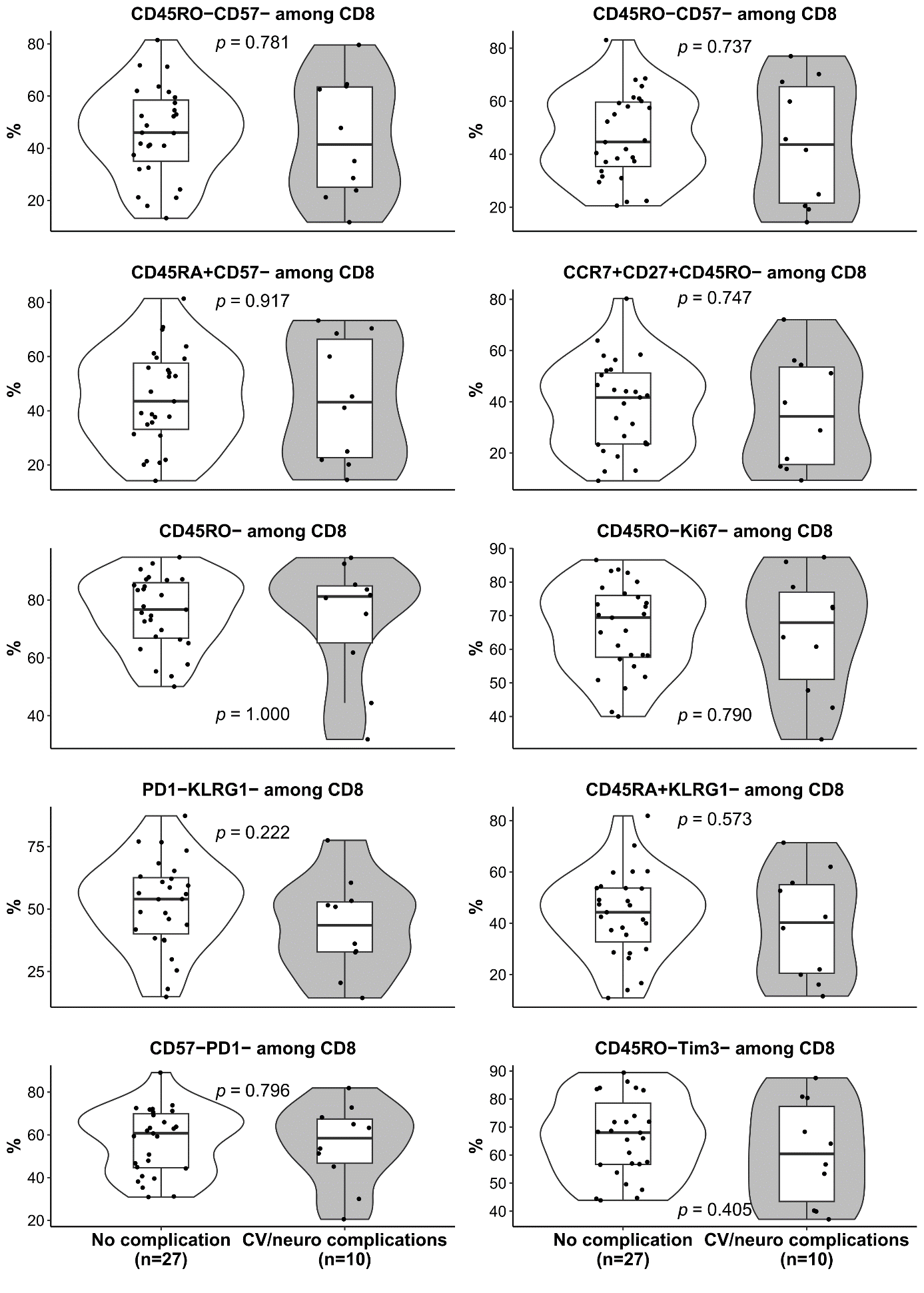


**A**

**
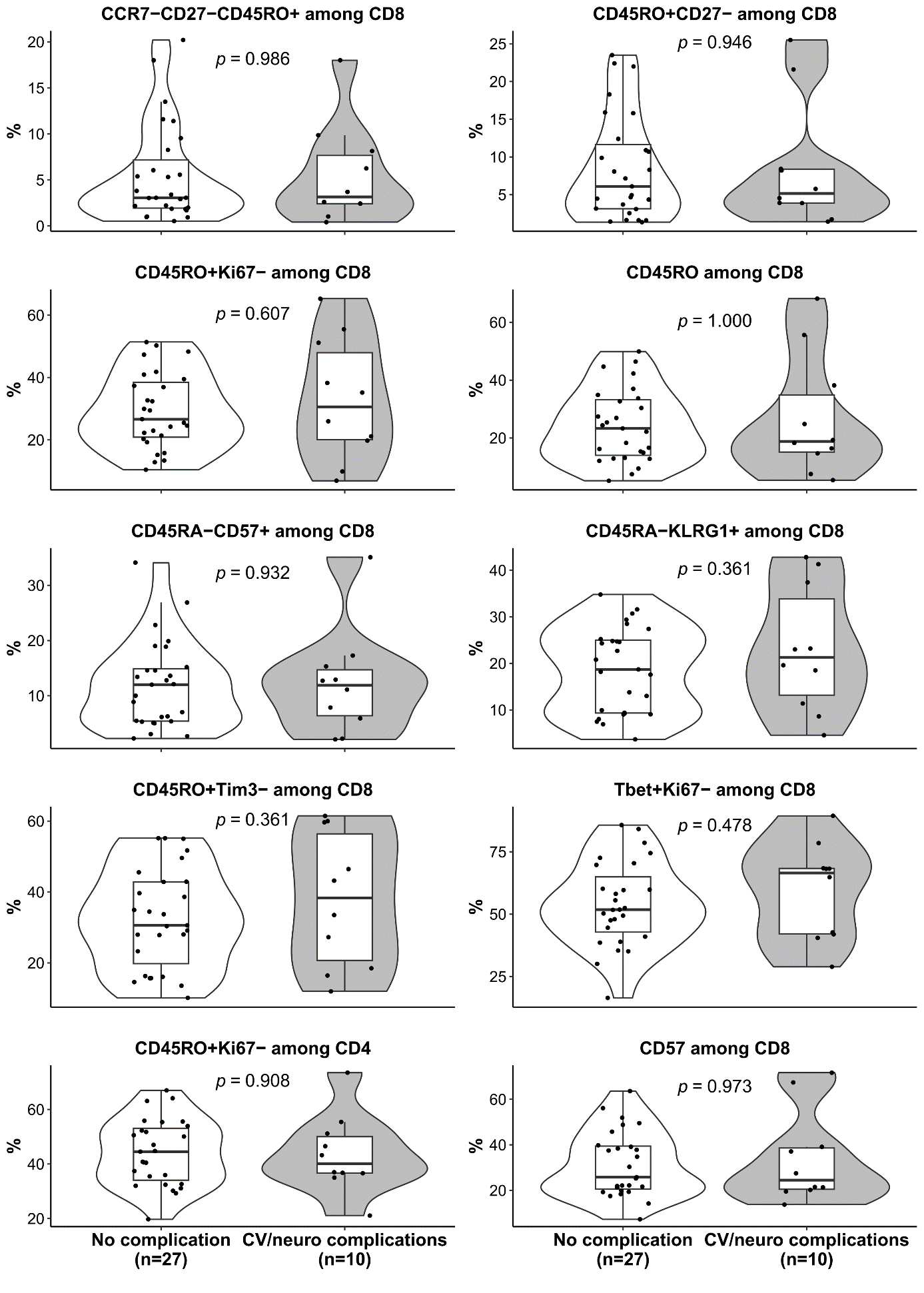
**

**B**

**Figure S2. Distribution of the immune features according to pot-COVID-19 complications.** All variables were recorded during acute COVID-19. Flow cytometry was performed on fresh PBMCs to investigate 484 cellular immune subsets or combinations of various lineage and functional markers. Panels show the distribution of each immune features from the top 10 positively (A) or negatively (B) correlated with LEF1-AS1 according to post-COVID-19 complications. . The 37 participants were divided into two groups: 10 participants with persistent cardiovascular and neurological complications 12 months after COVID-19 and 27 participants without complication. The Wilcoxon or T-test was used to compare continuous variables between the two groups. A *p*-value of <0.05 was considered significant.


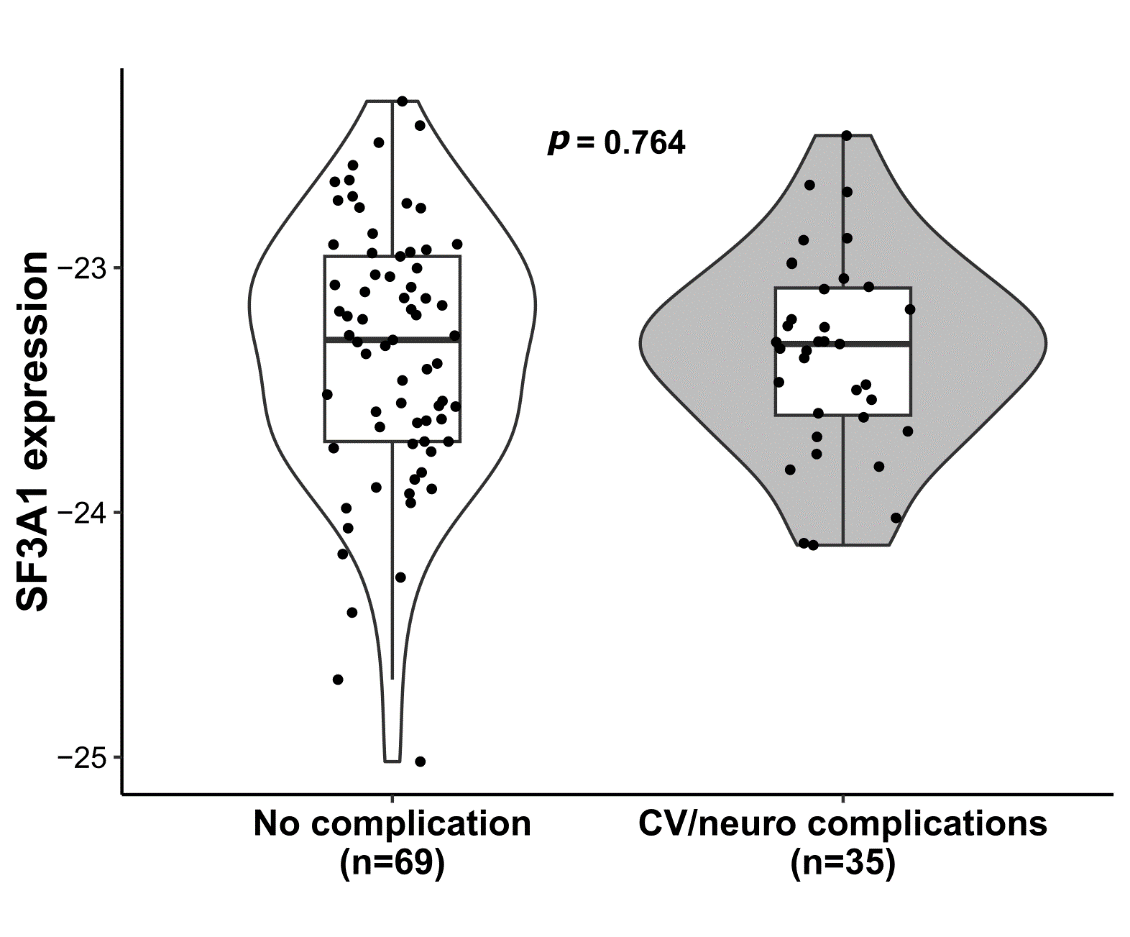


**Figure S3. SF3A1 expression according to post-COVID 19 complications.** The expression level of SF3A1 was measured by qPCR using total RNA extracted from PAXgene RNA blood tubes collected at baseline. SF3A1 expression was Log2 transformed. The 104 participants were divided into two groups: 35 participants with persistent cardiovascular and neurological complications 12 months after COVID-19 and 69 participants without complication. A Student’s t-test was used to compare the two groups.
